# *Wolbachia* confers protection against the entomopathogenic fungus *Metarhizium pingshaense* in African *Aedes aegypti*

**DOI:** 10.1111/1758-2229.13316

**Published:** 2024-08-04

**Authors:** Etienne Bilgo, Maria Vittoria Mancini, Jacques E. Gnambani, Houeffa Adeline Tatiana Dokpomiwa, Shivan Murdochy, Brian Lovett, Raymond St. Leger, Steven P. Sinkins, Abdoulaye Diabate

**Affiliations:** ^1^ Institut de Recherche en Sciences de la Santé Direction Régionale de l'Ouest Dioulasso Burkina Faso; ^2^ Institut National de Santé Publique/Centre Muraz Dioulasso Burkina Faso; ^3^ MRC‐University of Glasgow Centre for Virus Research Glasgow UK; ^4^ Department of Biology and Biotechnology University of Pavia Pavia Italy; ^5^ United States Department of Agriculture Research Service Ithaca New York USA; ^6^ University of Maryland College Park Maryland USA

## Abstract

Symbiotic and pathogenic microorganisms such as bacteria and fungi represent promising alternatives to chemical insecticides to respond to the rapid increase of insecticide resistance and vector‐borne disease outbreaks. This study investigated the interaction of two strains of *Wolbachia*, *w*AlbB and *w*Au, with the natural entomopathogenic fungi from Burkina Faso *Metarhizium pingshaense*, known to be lethal against *Anopheles* mosquitoes. In addition to showing the potential of *Metarhizium* against African *Aedes aegypti* wild‐type populations, our study shows that the *w*AlbB and *w*Au provide a protective advantage against entomopathogenic fungal infections. Compared to controls, fungal‐infected *w*Au and *w*AlbB‐carrying mosquitoes showed higher longevity, without any significant impact on fecundity and fertility phenotypes. This study provides new insights into the complex multipartite interaction among the mosquito host, the *Wolbachia* endosymbiont and the entomopathogenic fungus that might be employed to control mosquito populations. Future research should investigate the fitness costs of *Wolbachia*, as well as its spread and prevalence within mosquito populations. Additionally, evaluating the impact of *Wolbachia* on interventions involving *Metarhizium pingshaense* through laboratory and semi‐field population studies will provide valuable insights into the effectiveness of this combined approach.

## Graphical abstract

*Wolbachia* strains *w*AlbB and *w*Au can protect *Aedes aegypti* mosquitoes against infections from the entomopathogenic fungus *Metarhizium pingshaense* and this interaction could be utilized synergistically to develop novel Dengue mosquito control strategies.

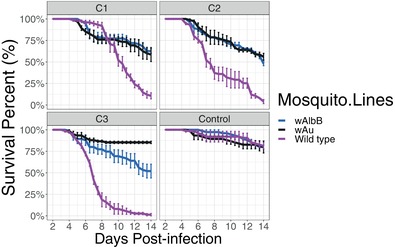

## INTRODUCTION

The burden of *Aedes*‐mosquito transmitted diseases represents one of the major global health challenges, with incidence increasing considerably over the past 50 years (Bhatt et al., 2013). Reported cases of dengue alone, the world's most common arboviral disease, increased over 8‐fold with a dramatic increment in reported deaths over the last decades (World Health Organisation, 2023). In addition to dengue, other arboviruses, such as Zika and chikungunya cause severe disease outbreaks in many urban environments, together with re‐emerging old threats, such as yellow fever (Espinal et al., 2019). Although the epidemiology of dengue in Africa has been less characterized than in other areas, laboratory‐confirmed official reports showed that dengue fever is present in 20 countries on the African continent. Nevertheless, a recent increase in the amount of dengue outbreaks was also registered in several West‐Central African countries, including Burkina Faso where a localized outbreak of dengue with 1061 probable cases was reported in 2016 (Ima et al., 2020). The primary vector of dengue in Africa is the mosquito *Aedes aegypti*, a highly anthropophilic daytime biter (Badolo et al., 2022).

The recent high incidence of dengue virus (DENV) infection in African countries can be attributed in part to improvements in diagnosis, but also to the increasing rate of urbanization in the continent, together with increases in international travel leading to imported cases. Urbanization is often correlated with increased availability of breeding sites for *Ae. aegypti* (Franco et al., 2003; Mwanyika et al., 2021) in regions with a warm and humid climate (Mourya et al., 2020; Sarfraz et al., 2019; Silva et al., 2021).

For the majority of mosquito‐borne viral diseases, there are currently no approved vaccines or specific antiviral therapies, and the efforts to fight most arboviruses rely on vector control strategies. Traditional methods aimed at decreasing population density with insecticides are increasingly ineffective. Recent concerning data in Burkina Faso revealed pyrethroid resistance and paired *kdr* mutations in both urban and semi‐urban sites at levels that are unprecedented for mainland Africa (Sombié et al., 2019; Sombié et al., 2023). The spread of insecticide resistance in *Ae. aegypti* has challenged the standard public health approaches to control those diseases; hence there is a real need for investment in developing new tools.

*Wolbachia‐*mediated strategies for dengue control show considerable promise due to the unique characteristics of *Wolbachia*. This bacterium is an intracellular, maternally inherited symbiont that impacts the vector dynamics of mosquitoes. Notably, *Wolbachia*‐carrying mosquitoes exhibit reduced susceptibility to a range of arboviruses, which includes dengue, lowering transmission rates (Ant et al., 2018; Frentiu et al., 2014; Moreira et al., 2009; Walker et al., 2011). Beyond this, *Wolbachia* can induce changes in mosquito fitness and reproductive patterns. A key phenomenon utilized in these control strategies is cytoplasmic incompatibility (CI), where *Wolbachia* affects the reproductive success of mosquitoes, leading to a reduction in the offspring of *Wolbachia*‐free females when they mate with *Wolbachia‐*carrying males, thereby promoting the spread of *Wolbachia* through mosquito populations (Hancock et al., 2011; Serbus et al., 2008; Werren et al., 2008).

Field trials and operational deployment of *Wolbachia*‐carrying *Ae. aegypti* are underway in many endemic regions of the world, offering the real prospect of significantly reducing the global disease burden caused by arboviruses (Ahmad et al., 2021; Utarini et al., 2021). One example is the *Wolbachia* strain *w*AlbB, native to *Aedes albopictus* and artificially transferred into *Ae. aegypti*. Open field releases launched in 2016 demonstrated the establishment of *w*AlbB‐*Ae. aegypti* at high frequency in wild populations within dengue hotspots in urban Kuala Lumpur, Malaysia, resulting in significant reductions in dengue incidence (Cheong et al., 2023). In laboratory‐based assays, the *w*AlbB‐line shows unidirectional CI, strong antiviral activity against major arboviruses and stable intracellular density under high rearing temperatures (Ant et al., 2018). Moreover, the originally generated *w*AlbB‐carrying line has been used recently for introgression and characterization in a Burkina Faso colony of *Ae. aegypti*, showing similar phenotypic stability and strong DENV blockage (Mancini et al., 2024).

Another *Wolbachia* transinfection is the *w*Au‐carrying line, generated by transferring cytoplasm from embryos of its native host, *Drosophila simulans*, to *Ae. aegypti*. *w*Au showed an excellent antiviral capacity against DENV, Zika (ZIKV) and Semliki Forest Virus (SFV) and relative stability at high temperatures (Ant et al., 2018). A recent study exploring *Wolbachia*‐mediated antiviral mechanisms highlighted that *w*Au shows unique features in its inhibition of arboviruses compared to other *Wolbachia* strains (Rainey et al., 2023). Assessment of the major life traits of *w*Au‐carrying mosquitoes indicated that this strain produces moderate costs on host fitness and is not able to manipulate host reproduction—notably, the CI factors, *cifA‐cifB* are absent in the *w*Au genome (Sutton et al., 2014). Nevertheless, *w*Au can spread and maintain high frequencies in natural populations of its native host, and cage experiments with flies demonstrated an increase in frequency from intermediate to fixation (Cao et al., 2019). Moreover, when flies were reared on food contaminated with fungal mycelia, *w*Au‐carrying females displayed larger size and increased fecundity, as well as extended developmental time at pupal stage (Cao et al., 2019). Although the contribution of *Wolbachia* to *Drosophila* fitness remains poorly understood and often indirectly demonstrated, it has been hypothesized that the ability to persist at high frequency is associated with the provision of beneficial traits and fitness advantages (Cao et al., 2019). Therefore, attributes of *Wolbachia* symbiosis and association with insect hosts are known to straddle between parasitism and mutualism: the selective benefits conferring advantageous fitness traits and ensuring maintenance in the population can also include protection from natural enemies.

Natural entomopathogenic fungi (EPF) are able to induce lethal epizootics on susceptible hosts. Due to their lethality, EPFs are used as agricultural biopesticides, and given their success, are now being explored for control of mosquito‐borne diseases. A field‐isolated strain of the lethal EPF, *Metarhizium pingahensae*, has been isolated from mosquitoes from Burkina Faso and found to be effective against *Anopheles coluzzii*, one of the major malaria vectors in West Africa. At low spore dosage, this strain showed strong virulence against *An. coluzzii* (LT_80_ of ~7 days), having minimal effects on non‐target insects such as honeybees and cockroaches (Bilgo et al., 2018).

Studies on the contribution of *Wolbachia* in protecting natural hosts against EPF are still limited. In *Drosophila melanogaster*, the naturally occurring strain *w*Mel provides some protection against *Beauveria bassiana* (Perlmutter et al., 2023), while no difference between survival rate of *Wolbachia*‐positive and *Wolbachia*‐negative populations of *D. simulans* was observed after infection with the same fungal strain (Cao et al., 2019). Natural *Wolbachia* infections do not protect spider mites from *B. bassiana* and *Metarhizium brunneum*, although variation between different populations was observed (Zélé et al., 2020). Artificial transinfections of *Wolbachia*, on the contrary, appeared to have a protective effect: wAlbB‐carrying *Ae. aegypti* survived longer than wild‐type mosquitoes when challenged with *B. bassiana* (Pan et al., 2018).

Our current study aims to test the pathogenic effects of the different local isolates of *M. pingshaense* on field‐collected *Ae. aegypti* from Burkina Faso, in comparison to other EPF. Moreover, we investigated whether two different *Wolbachia* strains, *w*AlbB and *w*Au, can confer protective advantages to mosquito larvae and adults against this fungal pathogen.

## EXPERIMENTAL PROCEDURES

### *Introgression of the* Wolbachia*‐carrying* Ae. aegypti *lines into an African genetic background*

The *Ae. aegypti* line carrying *Wolbachia w*Au was previously generated in a colonized wild‐type line from Selangor State, Malaysia (Ant et al., 2018). The G4 of the wild‐type field‐collected *Ae. aegypti aegypti* (BF_WT) from Bobo‐Dioulasso, Burkina Faso, was used for backcrossing wild‐type males with wAu‐carrying females for six consecutive generations. Cohorts of more than 500 adults were used for each backcross. *Wolbachia* transmission through generations was confirmed using end‐point PCR with strain‐specific primers for every generation of backcrossing, as described by Mancini et al., for the establishment of the wAlbB mosquito line (Mancini et al., 2024). Similarly to *w*Au, males of the wild‐type *Ae. aegypti aegypti* (BF_WT) were used for backcrosses with *w*AlbB‐carrying females for six consecutive generations.

BF_*w*AlbB and BF_*w*Au, together with the *Wolbachia*‐free BF_WT line, were maintained at 28°C, 70–80% RH, and a 12:12 h light: dark cycle, with ab libitum access to 5% sucrose solution. Females were fed using an artificial blood‐feeding system (Hemotek, UK) on human blood (Scottish National Blood Transfusion Service, UK). Eggs were collected on wet filter‐paper (Grade 1 filter paper, Whatman plc, GE healthcare, UK), desiccated for 5 days and hatched in deionized water containing 1 g/L bovine liver powder (MP Biomedicals, Santa Ana, CA). Larvae were maintained using tropical fish pellets (Tetramin, Tetra, Melle, Germany).

### Preparation of fungal strains used for bioassays

Bioassays were performed using native strains of *M. pingshaense* isolates and *Beauveria bassiana* from Burkina Faso (Bilgo et al., 2018). We used an atomizer protocol for infections, as described previously (Bilgo et al., 2018). Three serial concentrations were used: 1 × 10^8^, 1 × 10^7^ and 1 × 10^6^ conidia/ml. We confirmed that this inoculation technique was able to deliver a repeatable inoculating dose (mean ± SE): 276 ± 16 spores per mosquito with 1 × 10^8^, 211 ± 13 spores per mosquito with 1 × 10^7^ spores/ml; and 44 ± 3 spores per mosquito with 1 × 10^6^.

### Virulence and longevity assay on mosquito adults and larvae

Previous studies have indicated that *M. pingshaense* effectively kills mosquitoes at five spore doses (using an atomizer sprays) of 1 × 10^8^ spores/ml, 1 × 10^7^ spores/ml and 1 × 10^6^ spores/ml (Bilgo et al., 2017). A series of three doses starting from this effective diagnostic concentration was used to infect both *Wolbachia*‐carrying and non‐carrying *Ae. aegypti* mosquitoes. Mortality of fungus‐infected *Wolbachia*‐infected and non‐infected lines was monitored on a daily basis until all mosquitoes died. Five replicates of 50 mosquitoes/line/dose were used. In order to ascertain the cause of the death, mosquito cadavers were transferred to 1.5% agar plate to monitor the emergence of fungal hyphae.

### Wolbachia density

After the establishment of the introgressed line for assessing the phenotypic stability, the density of the *w*Au strain was quantified by qPCR. Whole bodies of 5‐day old G10 females were homogenized and genomic DNA was extracted by using STE buffer (10 μM Tris HCL pH 8, 100 mM NaCl, 1 mM EDTA). Quantitative analysis was performed by relative quantification of the *Wolbachia* surface protein (wsp) gene against the homothorax gene (*hth*) as a reference gene (Zélé et al., 2020). 2× SYBR‐Green Master Mix (Biotool, Houston, TX) with a BioRad CFX‐96 real‐time PCR detection system (Bio Rad, Hercules, CA) was used for the amplification reaction. The reaction was 95°C for 5 min, 40× cycles of 95°C for 15 s and 60°C for 30 s, followed by a melt‐curve analysis. In addition, *Wolbachia* density was also assessed on mosquito tissues. gDNA from five pools of three pairs of ovaries and salivary glands, and three midguts, were included for the analysis.

*Wolbachia* density was also investigated after exposure to *Metarhizium pingshaense*. A set of cadavers of mosquito killed by the entomopathogenic fungus after the bioassays were collected and the genomic DNA was extracted using 2% CTAB and diluted to a concentration of 100 ng/μl using a NanoDrop spectrophotometer (Thermo Scientific, MA). Bacterial density on whole mosquito bodies was quantified by using a qPCR detection system was used with the 2× PowerUp SYBR Green Master Mix kit (Thermo Scientific, MA). *w*Au and *w*AlbB relative quantification was performed by using strain‐specific primers annealing on the *Wolbachia wsp* gene (wAlbB [183F and qBrev2]; wAu [*w*AuF and *w*AuR]) in relation to the internal housekeeping control gene *hth* (Ant et al., 2018).

The following program was used to run the qPCRs: 50°C for 2 min, 95°C for 2 min, 40 cycles of 95°C for 15 s and 60°C for 1 min, followed by the melting curve analysis.

### Female fertility and fecundity

Thirty‐three‐day‐old inseminated females from each mosquito line (BF_WT mosquitoes, BF_*w*Au *Ae. aegypti*, and BF_*w*AlbB *Ae. aegypti*) were exposed to *Metarhizium pingshaense* at 1 × 10^6^ conidia/ml through spraying as described (Bilgo et al., 2018), while 0.05% Tween‐80 diluted in distilled water was used in the control treatment. For insemination, males and females from each line were permitted to mate in cages for 2 days prior to selecting the female mosquitoes for blood feeding on chickens. The females then received two blood meals at 48 and 72 h post‐insemination. Regarding blood feeding on chickens, the abdomens of the mosquitoes were checked to confirm that they were engorged with blood. Following the blood meals, each female was placed in an individual small cup lined with blotting paper. A layer of approximately 1 cm of tap water was added to the top of the paper to encourage egg laying and hatching. Fecundity was calculated as the average number of eggs laid/female, and the hatch rate was determined as the number of 2nd instar larvae per total number of eggs hatched.

## RESULTS

### *Entomopathogenic effects of native strains of* Metarhizium pingshaense *and* Beauveria bassiana *on* Ae. aegypti

**FIGURE 1:**
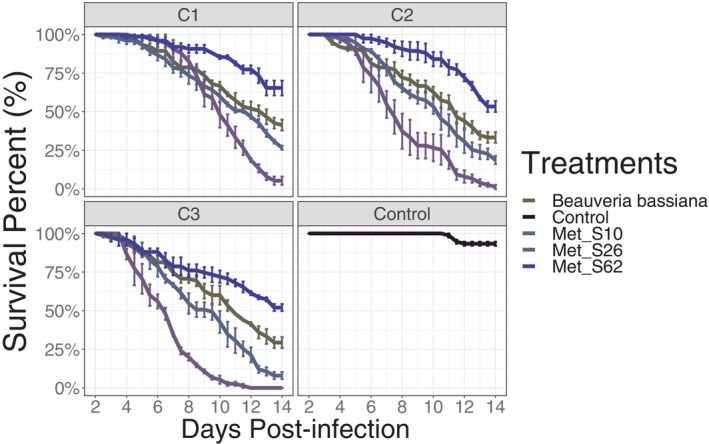
Survival curves of *Ae. aegypti* after exposure to native *Metarhizium pingshaense* strains and *Beauveria bassiana* from Burkina Faso at three different concentrations: C1, 1 × 10^6^ conidia/ml; C2, 1 × 10^7^ conidia/ml; C3, 1 × 10^8^ conidia/ml. Curves show the percentage survival with error bars indicating 95% confidence intervals from three replicate cages for each line, each containing a starting number of 25 adult females.

To determine the virulence of the different fungal isolates on a Burkina Faso population of *Ae. aegypti*, the survival percentage of adult mosquitoes was assessed after exposure to local isolates of *M. pingshaense* and *B. bassiana*. Mosquitoes were exposed to three different concentrations of fungal species (10^8^, 10^7^ and 10^6^ conidia/ml), while a control group was sprayed with a mock solution, containing only the suspension buffer. The survival curves of the infection of *Ae. aegypti* with three different fungal concentrations showed a dose dependent virulence for all strains. The higher dose resulted in the lowest survival over 14 days with (mean ± SE): 41.33 ± 3%, 26.67 ± 1.33%, 5.33 ± 3.3% and 65.33 ± 5.33% for *Beauveria bassiana*, Met‐S10, Met‐S26 and Met‐S62, respectively (Figure 1). Consistently between concentrations, only the *Metarhizium pingshaense* strain Met‐S26 achieved 80% mortality, showing higher virulence compared to the other strains tested (LT80 of 5.33 ± 3.3% days). The overall survival curve on the control group averaged 93.33 ± 1.3% during the bioassay (Figure 1).

### *Wolbachia‐conferred protection of* Ae. aegypti *against* M. pingshaense

#### Establishment of a wAu introgressed line in an African genetic background

The *w*Au *Wolbachia*‐carrying line, originally generated in a lab‐adapted Asian genetic background, was stably introgressed into a field‐collected African *Ae. aegypti* population from Burkina Faso. Mosquito backcrossing and introgression into local genetic backgrounds represent a crucial step for developing effective field‐applied mosquito control strategies.

In order to initially assess the interaction between the *Wolbachia* strain and the host genotype we quantified the symbiont intracellular density in whole bodies of females at 5 and 10 days post‐eclosion (DPE) Figure S1. In addition, in order to investigate the symbiont tropism, mosquito midguts, ovaries and salivary glands were dissected and assessed for tissue‐specific *Wolbachi*a density. Consistent with previous observations in other genetic backgrounds, a difference in intracellular density of *Wolbachia* was observed between tissues (*p* < 0.0001, Kruskal–Wallis), with ovaries and salivary glands having higher *Wolbachia* loads, compared to midguts (Figure S1).

### Impact of the fungal infection on adult survival

**FIGURE 2:**
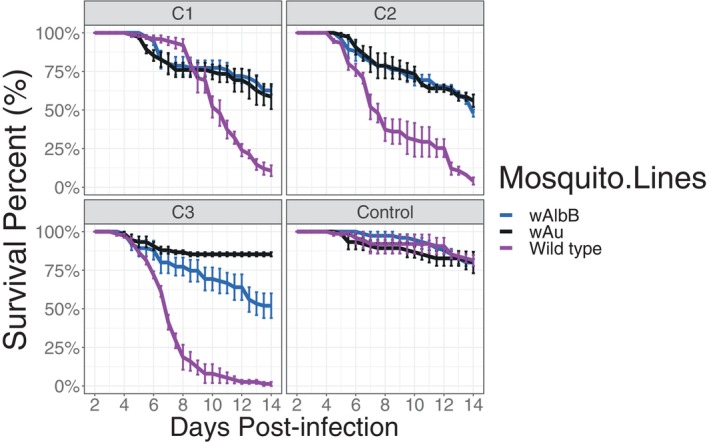
Survival curves for adult *Ae. aegypti* mosquitoes, BF_WT, BF_*w*AlbB and BF_*w*Au, after infection with *M. pingshaense* isolates Met‐S26 at different concentrations: C1, 1 × 10^6^ conidia/ml; C2, 1 × 10^7^ conidia/ml; C3, 1 × 10^8^ conidia/ml. Curves show the percentage survival with error bars indicating 95% confidence intervals from three replicate cages for each line each containing a starting number of 25 adult females.

The fungal strain Met‐S26 showing higher virulence against *Ae. aegypti* wild‐type was used for infecting BF_WT, BF_*w*AlbB and BF_*w*Au *Ae. aegypti* females. The survival of mosquitoes after fungal infection was monitored daily. The results demonstrate significantly improved survival of *Wolbachia*‐carrying lines compared to the wild‐type group, suggesting a symbiont‐conferred protection against the *Metarhizium* isolate, independently of fungal concentrations (Mantel‐Cox log‐rank test, *p* < 0.001). The strength of protection was similar between the two *Wolbachia* strains when mosquitoes were exposed to 10^6^ and 10^7^ fungal conidia/ml. However, when a higher concentration of entomopathogenic fungus was delivered (10^8^ fungal conidia/ml), the *w*Au strain was able to provide stronger protection compared to *w*AlbB. In terms of lethal doses, data analysis indicated differences in LT50 values between mosquitoes when infected with *M. pingshaense*, suggesting a difference in susceptibility among the three mosquito populations (Figure 2 and Table 1).

**TABLE 1:** LT50s and grouping LT50 values for of *Aedes aegypti* mosquitoes from the co‐infection *Wolbachia* (Strains: wAlbB and wAu) and Burkina Faso *Metarhizium pingshaense* isolate at three different spore concentrations.

Treatments (conidia/ml)	Mosquito strains	LT 50 + SE (days)	Grouping LT50b
C1 (1 × 10^6^)	*w*Au	13.0	a
	Wild type	10.5 ± 0.29	b
C2 (1 × 10^7^)	*w*AlbB	14.0	a
	Wild type	8.5 ± 1.05	b
C3 (1 × 10^8^)	*w*AlbB	12.5	a
	Wild type	7.0	b

*Note*: (a) In 0.01% Tween80. (b) Pairwise t‐test comparison of LT50 values per spraying conidia suspension concentrations; treatments with no letters in common differ significantly at *p* < 0.05. The missing mosquito strains for each treatment (conidia/ml) did not allow for the calculation of LT50 values and statistical comparisons.

### Impact of the fungal infection on larval survival

**FIGURE 3:**
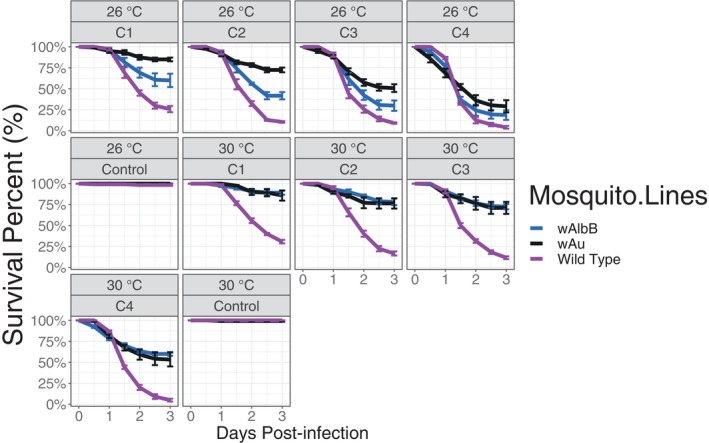
Survival curves of larval stages of *Ae. aegypti* mosquitoes, BF_WT, BF_*w*AlbB and BF_*w*Au at two temperature ranges (26°C and 30°C) after infection with *M. pingshaense* isolates Met‐S26 at different concentrations: C1, 1 × 10^4^ conidia/ml; C2,1 × 10^5^ conidia/ml; C3, 1 × 10^6^ conidia/ml and C4, 1 × 10^7^ conidia/ml. Curves show percentage survival with error bars indicating 95% confidence intervals from four replicate cages for each line each containing a starting number of 25 adult females.

The entomopathogenic effect of Met‐S26 and the protective phenotype of *Wolbachia* were also explored at mosquito larval stages, after exposure to several fungal concentrations at two different larval rearing temperatures (26°C and 30°C). Substantial virulence of the fungal isolate at different concentrations was observed on wild‐type, *Wolbachia*‐free mosquitoes for both environmental conditions. In contrast, *Wolbachia‐*carrying mosquitoes showed significantly reduced susceptibility to the fungus even at high fungal dosages (*p* < 0.001). However, significantly higher protection was achieved when larvae were reared at 30°C. At 26°C, *w*Au mosquitoes had significantly higher survival compared to *w*AlbB mosquitoes at the lowest fungus concentration (10^4^ conidia/ml). In addition, *Wolbachia*‐mediated protection from fungal infection was enhanced at 30°C for both *Wolbachia* strains, with average survival dropping below 50% only in the highest tested concentration (10^7^ conidia/ml; Figure 3). At this highest concentration, survival was near or at zero, regardless of temperature or *Wolbachia* symbiont status (Figure 3).

### Wolbachia density after exposure to fungal infections in adults

**FIGURE 4:**
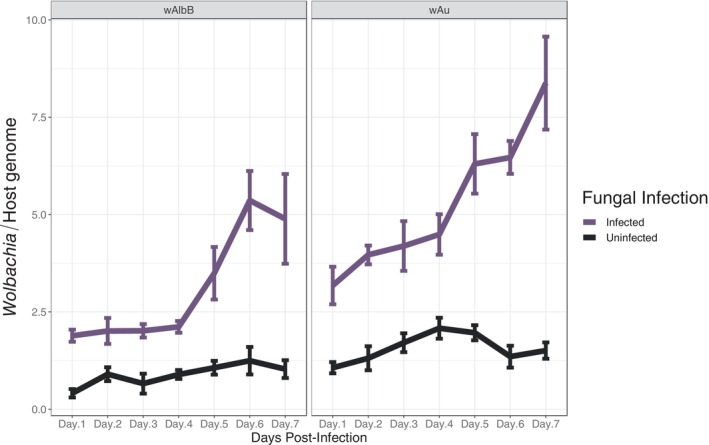
Impact of fungal infection Met‐S26 on *w*AlbB and *w*Au density in adult *Ae. aegypti* mosquitoes at different time points following fungal infections. *Wolbachia* density was determined from days 1 to 7 after fungal exposure using qPCR (*N* = 42). Points on the line show median with error bars indicating 95% confidence intervals or SE.

By assessing *Wolbachia* density after fungal co‐infections, we aimed at exploring potential perturbations of the symbiont when a pathogenic agent was present. In the control groups, the mean density of *Wolbachia* over a 7‐day observation period was higher in *w*Au mosquitoes (1.57 ± 0.1 Δ/Δ Ct16S Wolbachia/HTH) compared to *w*AlbB mosquitoes (0.89 ± 0.09 Δ/Δ Ct 16S Wolbachia/HTH) (Welch Two Sample *t*‐test, *t* = −5.1972, df = 80.267, *p* value <0.001), as shown Figure 4. After *M. pingshaense* infection, the density of *Wolbachia* gradually increased. For the *w*AlbB strain, the density increased significantly from approximately 4.61 fold on day 1 post‐infection to approximately 4.75 times on day 7 post‐infection with fungi, compared to uninfected *w*AlbB strains on the same days post infection (Figure 4). Similarly, *w*Au density significantly increased over time, ranging from 3 times on the first day post‐fungal infection and reaching an average of around 5.56 times on day 7 post‐fungal infection (Figure 4).

### Female fitness after fungal infections

**FIGURE 5:**
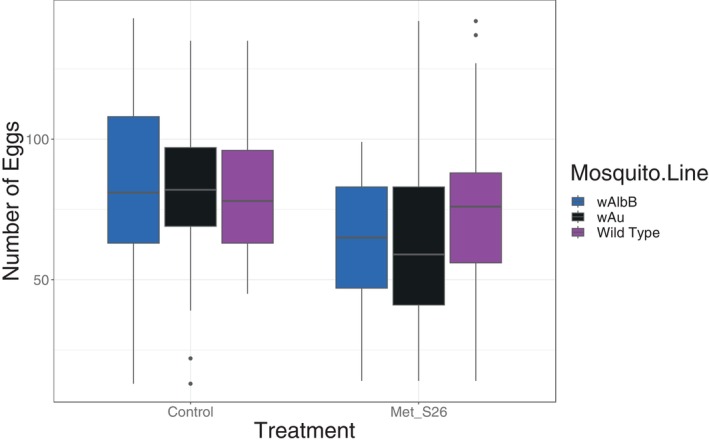
The effect of co‐infection with fungi (*Metarhizium pingshaense* Met_S26) and *Wolbachia* on the number of eggs laid per female mosquito carrying *Wolbachia* (*w*Au and *w*AlbB) versus wild *Aedes aegypti* not carrying *Wolbachia*.

**FIGURE 6:**
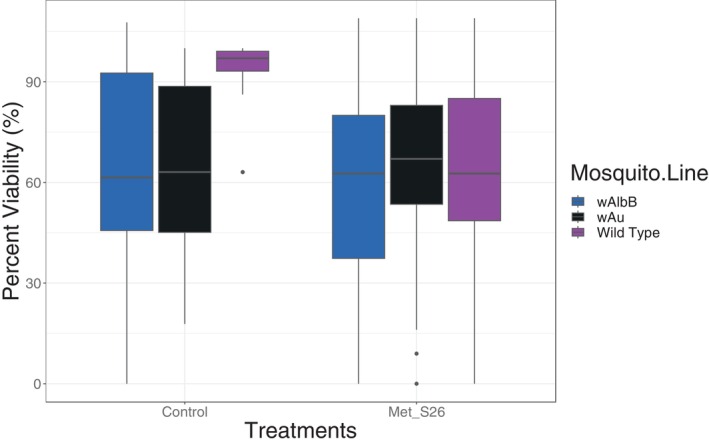
The effect of co‐infection with fungi (*Metarhizium pingshaense* Met_S26) and *Wolbachia* on the viability of eggs of mosquito carrying *Wolbachia* (*w*Au and *w*AlbB) versus wild *Aedes aegypti* not carrying *Wolbachia*. Viability is the mean number of eggs hatching and reaching the larval stage L1‐L2.

The Figure 5 presents the results of the impact of fungal infection on *Wolbachia*‐carrying females compared to the wild‐type, focusing on the number of eggs laid per mosquito. Single uninfected BF_WT, BF_*w*AlbB, and BF_*w*Au produced an average number of eggs of 80.78 ± 3.52, 81.56 ± 3.4 and 83 ± 4.69, respectively. For the control treatment, the Tukey post‐hoc test was conducted to compare the mean number of eggs laid by mosquitoes across different mosquito lines. The comparisons between the mosquito lines did not reveal any significant differences. Specifically, the differences between *w*Au and *w*AlbB (Estimate = 8.976, SE = 11.318, *t* = 0.793, *p* = 0.707), Wild Type and *w*AlbB (Estimate = 14.464, SE = 10.387, *t* = 1.393, *p* = 0.345), and Wild Type and *w*Au (Estimate = 5.489, SE = 10.048, *t* = 0.546, *p* = 0.848) were all non‐significant. Regarding the *Metarhizium pingshaense* treated mosquitoes; the Tukey post‐hoc test was conducted to compare the mean number of eggs laid by mosquitoes across different mosquito lines. The results showed no significant differences between the mosquito lines. Specifically, the comparisons between *w*Au and *w*AlbB (Estimate = −11.09, SE = 15.11, *t* = −0.734, *p* = 0.742), Wild Type and *w*AlbB (Estimate = 11.23, SE = 13.08, *t* = 0.859, *p* = 0.666), and Wild Type and *w*Au (Estimate = 22.32, SE = 13.08, *t* = 1.706, *p* = 0.204) were all non‐significant. However, the Figure 6 shows a decrease in viability, measured as the number of eggs that hatched and reached the larval stage, between fungal treated and control mosquitoes. BF_WT showed a 95.51% hatching rate, while *Wolbachia*‐infected mosquitoes (*w*Au and *w*AlbB) displayed hatching rates of 66.02% and 67.32%, respectively. The results show a significant decrease in vitality for the *Metarhizium pingshaense* treatment compared to the control (Estimate = −42.08, SE = 13.85, *t* = −3.039, *p* = 0.00258).

## DISCUSSION

In this study, the virulence of native strains of EPF *M. pingshaense* from Burkina Faso, previously assessed only on *Anopheles coluzzii*, has been also demonstrated on a recently field‐collected Burkina Faso population of *Ae. aegypti*. In particular, the strain Met‐S26, a natural isolate of *Metarhizium*, (Bilgo et al., 2018), showed strong pathogenicity also against *Ae. aegypti* Met‐S26 induced 80% mortality in the *Ae. aegypti* populations (LT80) even at low dosages, which meets the threshold value set by the guidelines of the World Health Organization Pesticide Evaluation Scheme (WHOPES) for scoring an insecticide‐based intervention as successful (World Health Organization, 2013).

In addition, we showed that the artificial transinfections of the *Wolbachia* strains *w*AlbB and *w*Au in populations of *Ae. aegypti* from Burkina Faso protected mosquitoes from the pathogenic effects of the fungal isolate, increasing their survival rate. Although both *Wolbachia* strains provided significant protection, the effect was stronger in *w*Au‐carrying individuals, also at very high fungal dosages. This is in line with previous evidence on *w*Au ability to provide strong inhibition against arboviruses and further supports the hypothesis that *w*Au high intracellular density plays a role in or least contributes to conferring protective phenotypes. This protection could provide a strong selective advantage for *Wolbachia*‐carriers compared to their *Wolbachia*‐free counterparts where *M. pingshaense* are naturally abundant or are deployed.

In the case of the *w*Au strain, which lacks the ability to cause advantageous reproductive manipulations like CI, it shows promising results for potential population replacement strategies. Due to its strong blockage capacity, several strategies have been theorized in order to convert *w*Au into a self‐sustainable and invasive strain. The identification of the CI‐inducing genes, *cifA* and *cifB* genes, offers the possibility of integrating CI‐carrying elements into *w*Au genome, in which they are known to be absent. Alternatively, the combination of *w*Au with a driving element could be explored for exploiting the promising features of this strain. For instance, a *Wolbachia* strain capable of causing CI could drive both infections into an uninfected population. As a proof of concept, a *w*Au*w*AlbB line was produced, showing full unidirectional CI when crossed with wild‐type mosquitoes. Although reaching a very similar intracellular density to *w*Au as a single infection, the fitness effects on the major life history traits of the superinfected line still need to be fully characterized and its field application potential elucidated (Ant et al., 2018; Rainey et al., 2023).

Now, the evidence of *Wolbachia* strains to provide strong selective traits to the hosts could be a driving system for the spread of strains lacking reproductive advantages (i.e., *w*Au) or for improving and promoting the invasion of CI‐inducing strains, like *w*AlbB. As observed during field release trials of *w*AlbB‐carrying mosquitoes in Kuala Lumpur, areas with low population density, weak geographical isolation barriers or frequent immigration of *Wolbachia*‐free mosquitoes from outside release areas, can reduce *Wolbachia* frequency and impact on control interventions (Cheong et al., 2023). The selection of EPF‐resistant *Wolbachia*‐carrying populations could then be exploited for improving its spread and stability, by using a synergistic strategy integrating fungal‐based interventions and *Wolbachia* deployment approaches for potentially improving sustainable control of dengue.

A modelling study investigating *Wolbachia* transmission dynamics demonstrated that *w*Au high transmission fidelity and high viral blockage can compensate for the lack of CI and has the potential to establish the conditions for local stability, if specific conditions are considered, such as continuous introduction at a larger scale of *w*Au‐carrying mosquitoes (Ogunlade et al., 2020). Here, we found an extra selective advantage, that the presence of the entomopathogenic fungus could improve this prediction, by acting as a driver for *w*Au invasion. Specifically, in a mixed population of mosquitoes (i.e., some carrying *w*Au and some not), our results suggest the impact of the fungal pathogen will be uneven, particularly at the larval stage under field‐relevant high temperatures. When challenged by a fungal pathogen, this differential mortality would lead to more adults carrying *w*Au with each generation, with overall effectiveness of the entomopathogen decreasing as the proportion of *w*Au carrying mosquitoes increases over time. Many factors may affect this interaction, including our observed decrease in egg production and hatching rate of *Wolbachia*‐carrying *Ae. aegypti* and further decrease in fertility during fungal infection.

The impact of *Metarhizium* entomopathogenic infection extends to the intracellular density of both *w*AlbB and *w*Au strains, particularly evident in the case of the *w*Au strain. This increase in overall density would potentially contribute to the enhanced ability of *Wolbachia*‐carrying mosquitoes to inhibit virus transmission, as density is typically positively correlated with virus blockage (Ye et al., 2013). However, this advantage could be outweighed if the observed increase in bacterial density also induces a significant increase in fitness cost for the host. Limited data are available regarding *Wolbachia* density perturbations following pathogen infections in mosquitoes. In a previous study that assessed *Wolbachia* density after exposure to various *Beauveria* species, divergent results were observed (Ramirez et al., 2021). A decrease in density was observed in native *Wolbachia* strains of *Ae. albopictus*, whereas no effects were observed in naturally occurring strains in *Cule*x (Ramirez et al., 2021). The discrepancy in outcomes can be attributed to the substantial and well‐established differences in host phenotypes conferred by *Wolbachia* between artificial (BF_*w*AlbB and BF_*w*Au) and natural infections of this symbiont. Artificial transinfections, characterized by stronger pathogen blockage, also tend to exhibit higher *Wolbachia* density, as well as distinct tissue tropism and localization (Joubert et al., 2016; Rainey et al., 2023).

The presence of *Wolbachia* bacteria in mosquitoes can have various effects on their reproductive biology, including reducing egg hatch following egg quiescence (dormancy in a dry state) and reduced fecundity of the resulting females (Ant et al., 2018; Axford et al., 2016; Lau et al., 2021). However, the impact of co‐infection with *Wolbachia* and fungal infections on egg hatch is not yet fully understood. There will be complex interactions between *Wolbachia* strain and the fungal infection, as well as additional factors including environmental conditions and the specific mosquito species under investigation. Research on the effects of co‐infection with *Wolbachia* and fungi on fertility is still limited. Some studies have reported that co‐infection can have a synergistic effect on reducing mosquito fecundity (Jousset et al., 2017; Mains et al., 2018). On the other hand, other studies have suggested that the presence of *Wolbachia* may confer some protection against fungal infections, leading to minimal or no impact on fertility (Zélé et al., 2014). The complexity of the interactions between *Wolbachia* and fungal infections can be attributed to multiple factors. Firstly, the specific strains of *Wolbachia* and the fungal pathogens involved can greatly influence the outcome. Different strains of *Wolbachia* may have varying effects on mosquito biology, including their reproductive capacity (Ross et al., 2020). Similarly, the virulence and pathogenicity of fungal infections can differ depending on the species and strains involved (Zélé et al., 2014). Additionally, environmental conditions play a crucial role in shaping the outcome of co‐infections. Temperature, humidity, and other ecological factors can impact the growth and survival of both *Wolbachia* and fungal pathogens, ultimately affecting their interactions within the mosquito host (Mancini et al., 2021; Ross et al., 2020). Furthermore, the mosquito species being studied can have distinct physiological and immunological responses to co‐infection, which may further influence their reproductive biology (Ramirez et al., 2021).

*Wolbachia*‐conferred resistance to *Metarhizium* has promising potential applications in the frame of biological control strategies for *Aedes* mosquitoes. A comparative fitness benefit associated with *Wolbachia* infection has been observed, but the influence of exposure to entomopathogenic fungi on pathogen blocking or the ability of *Wolbachia* to invade populations remains unknown and needs to be investigated. Research is also needed on the mechanistic basis of the protection conferred by *Wolbachia* against entomopathogenic fungi. Additionally, future studies should examine how fitness costs, such as decreased longevity and potential increases in *Wolbachia* density, might affect the spread and prevalence of *Wolbachia* within populations. In the same vein, to fully evaluate the effectiveness of this approach, invasion population studies in lab settings and semi‐field conditions could offer more comprehensive results. Exploring how *Wolbachia* could impact interventions involving *Metarhizium* spp., especially if similar protective effects occur in target mosquito species naturally infected by *Wolbachia*, would provide valuable insights. Lastly, for the development of this combined strategy, a pathway for development needs to be put in place considering efficacy, safety, regulation, and socio‐economic considerations throughout the process.

The demonstration of the susceptibility of the field‐collected wild‐type population of *Ae. aegypti* to the entomopathogenic *Metarhizium* not only provides an additional tool for *Ae. aegypti* control using natural bio‐control agents, but it also offers the opportunity for a synergistic approach for enhancing *Wolbachia*‐based strategies. By harnessing the selective protective advantage conferred by *Wolbachia* against the entomopathogen, a driving force for improving *Wolbachia* invasions dynamics could be applied. This aspect becomes particularly impactful when *Wolbachia* strains with excellent antipathogenic potential, but limited ability to spread through mosquito populations, are considered for field deployment.

## AUTHOR CONTRIBUTIONS

**Etienne Bilgo:** Conceptualization; investigation; writing – original draft; methodology; validation; visualization; writing – review and editing; software; formal analysis; data curation; supervision; resources. **Maria Vittoria Mancini:** Conceptualization; investigation; writing – original draft; methodology; validation; visualization; writing – review and editing; software; data curation. **Jacques E. Gnambani:** Conceptualization; methodology; validation; writing – review and editing; visualization; data curation. **Houeffa Adeline Tatiana Dokpomiwa:** Conceptualization; methodology; validation; visualization; writing – review and editing; software; data curation. **Shivan Murdochy:** Investigation; methodology; validation; writing – review and editing; software; data curation. **Brian Lovett:** Investigation; writing – review and editing; validation; visualization; supervision. **Raymond St. Leger:** Investigation; writing – review and editing; methodology; validation; supervision. **Steven P. Sinkins:** Conceptualization; investigation; funding acquisition; writing – review and editing; methodology; validation; visualization; project administration; software; formal analysis; supervision. **Abdoulaye Diabate:** Conceptualization; investigation; funding acquisition; writing – review and editing; visualization; validation; methodology; supervision; project administration; formal analysis; software; resources.

## CONFLICT OF INTEREST STATEMENT

The authors declare no conflict of interests.

## ETHICS STATEMENT

Ethical permissions required for this study were obtained through the Institutional Review of Institut de Recherche en Science de la Sante' (IRSS) and Centre Muraz ethics committee.

## Supporting information


**FIGURE S1.** Wolbachia density in whole bodies (A) and dissected organs (B) in the introgressed BF_wAu line after six backcrossings. Wolbachia density was determined at 5 and 10 days post‐adult eclosion by qPCR (*N* = 12). Five biological replicates of three sets of salivary glands, midguts and ovaries from 5‐days old females were analysed. Boxplots show median and interquartile ranges. Ovaries and salivary glands have higher Wolbachia loads, compared to midguts (*p* < 0.0001, Kruskal–Wallis).

## Data Availability

The datasets generated during the current study, R code (scripts) for all statistical analysis and visualizations (graphs) are available in this GitHub repository. https://github.com/EtienneBilgo/Bilgo‐et‐al_Environmental‐Microbiology‐Reports‐/upload/main.
