# Supplementary material for: Wolbachia confers protection against the entomopathogenic fungus Metarhizium pingshaense in African Aedes aegypti
Source: Environ Microbiol Rep. 2024 Aug 4;16(4):e13316. doi: 10.1111/1758-2229.13316 (PMC11298244; doi:10.1111/1758-2229.13316)
Supplement: Supplementary file 1 — FIGURE S1. Wolbachia density in whole bodies (A) and dissected organs (B) in the introgressed BF_wAu line after six backcrossings. Wolbachia density was determined at 5 and 10 days post‐adult eclosion by qPCR (N = 12). Five biological replicates of three sets of salivary glands, midguts and ovaries from 5‐days old females were analysed. Boxplots show median and interquartile ranges. Ovaries and salivary glands have higher Wolbachia loads, compared to midguts (p < 0.0001, Kruskal–Wallis). [file EMI4-16-e13316-s001.docx]

A B

*
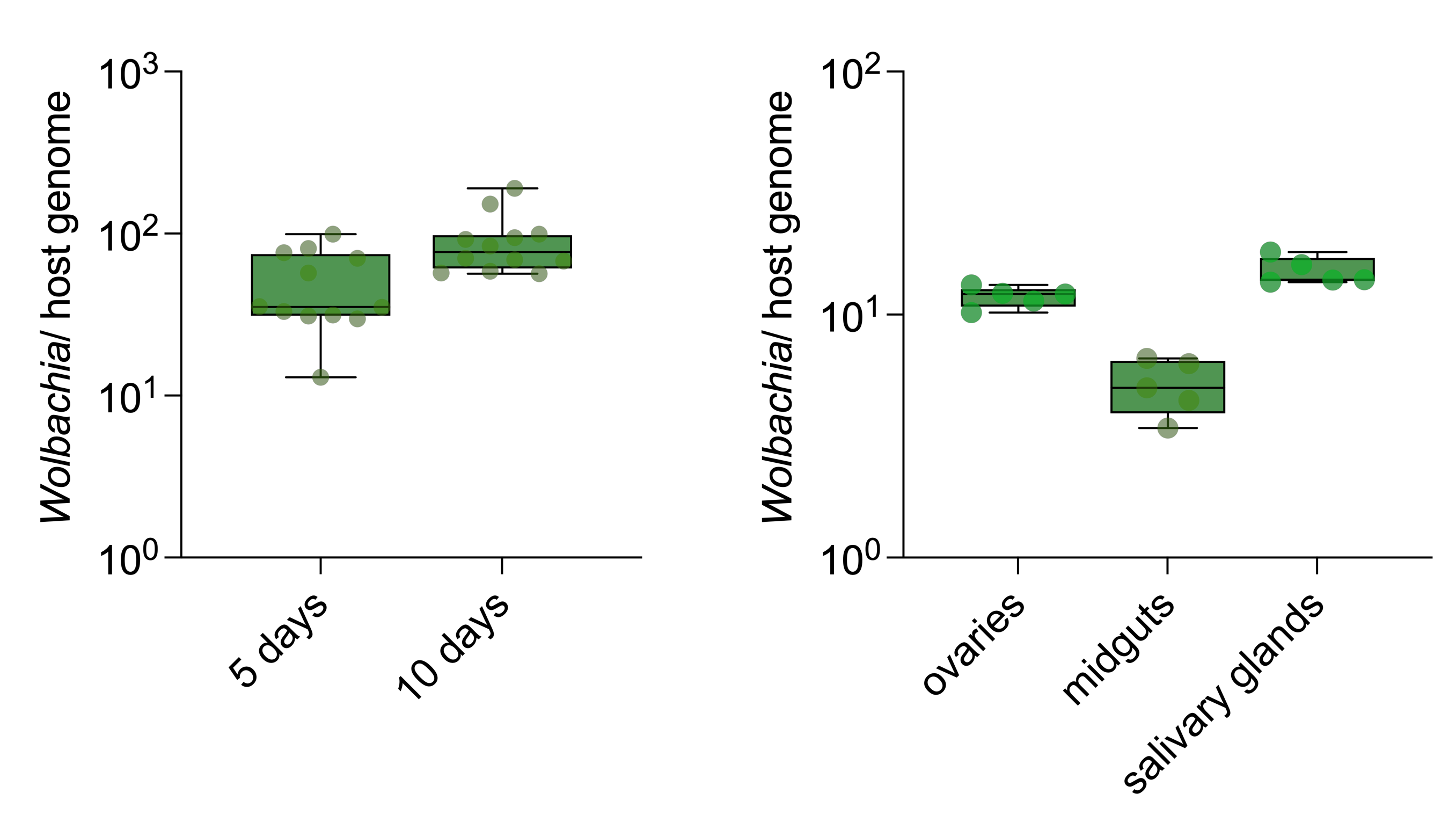
*

Figure S1: Wolbachia density in whole bodies (A) and dissected organs (B) in the introgressed BF_wAu line after 6 backcrossings. Wolbachia density was determined at 5 and 10 days post-adult eclosion by qPCR (N=12). Five biological replicates of three sets of salivary glands, midguts and ovaries from 5-days old females were analyzed. Boxplots show median and interquartile ranges. Ovaries and salivary glands have higher Wolbachia loads, compared to midguts (p<0.0001, Kruskal-Wallis).
